# The role of the beta cell in type 2 diabetes: new findings from the last 5 years

**DOI:** 10.1007/s00125-025-06499-z

**Published:** 2025-08-06

**Authors:** Belinda Yau, Julien Ghislain, Melkam A. Kebede, Jing Hughes, Vincent Poitout

**Affiliations:** 1 Charles Perkins Centre, University of Sydney, Camperdown, NSW Australia; 2 School of Medical Sciences, Faculty of Medicine and Health, University of Sydney, Camperdown, NSW Australia; 3 University of Montreal Hospital Research Center, Montreal, QC Canada; 4 Department of Medicine, Yale University School of Medicine, New Haven, CT USA; 5 Department of Medicine, University of Montreal, Montreal, QC Canada

**Keywords:** Human islets, Pancreatic beta cell, Remission, Review, Type 2 diabetes

## Abstract

Recent advances in genome-wide approaches, the availability of isolated human islets for research and the evaluation of novel incretin mimetics in large clinical trials have brought about remarkable progress in our understanding of the role of the pancreatic beta cell in type 2 diabetes. Here, we review key developments in type 2 diabetes initiation, progression and remission, focusing mostly on human studies published in the last 5 years. Progress in multi-omics technologies has enabled researchers to identify links between type 2 diabetes risk variants and gene regulatory networks in islet endocrine cells that control beta cell development, function and stress resilience. These studies support the notion that early abnormalities in insulin secretion, rather than a reduction in beta cell mass, play a fundamental and primary role in early type 2 diabetes pathogenesis. Contributing to these intrinsic beta cell defects are various pathogenic signals from other (endocrine and non-endocrine) islet cells, the exocrine pancreas, the gut and insulin-sensitive tissues. It has also become apparent that beta cells comprise a heterogeneous population that responds differently to stress situations and that sex-related differences in beta cell responses should not be underestimated. Finally, human clinical trials have clearly demonstrated that diabetes remission can be achieved using glucose-lowering therapies and particularly strategies focused on weight loss, including bariatric surgery and, more recently, the use of highly efficient new drugs targeting the incretin system. While progress in the last 5 years has been significant, much remains to be uncovered to bring these advances to the clinic and thereby alleviate the dramatic consequences of type 2 diabetes complications for the hundreds of millions of people who live with this disease.

**Graphical Abstract:**

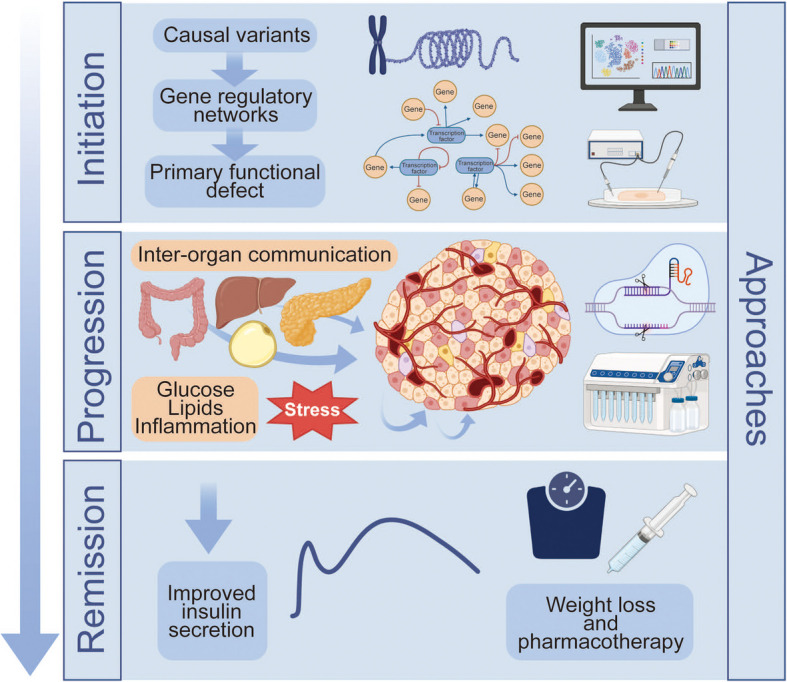

**Supplementary Information:**

The online version contains a slide of the figure for download available at 10.1007/s00125-025-06499-z.

## Introduction

The pancreatic beta cell plays a key role in the maintenance of glucose homeostasis by regulating insulin secretion in response to metabolic cues. In type 2 diabetes, beta cell dysfunction is a key early pathogenic event that contributes to disease onset and progression. Conversely, improvements in beta cell function are linked to type 2 diabetes remission, underscoring the critical role of beta cells in both the deterioration and the potential recovery of glucose regulation. The objective of this review is to summarise the recent developments in our understanding of the role of the beta cell in the early pathogenesis, progression and remission of type 2 diabetes. We focus specifically on recent knowledge gained from human islet biology and pathophysiology and mention rodent studies only when they directly inform human disease pathogenesis. Likewise, we emphasise publications from the last 5 years and cite earlier studies when necessary to contextualise recent work. We apologise to authors of relevant studies that could not be cited because of space limitations.

## The role of the beta cell in early type 2 diabetes pathogenesis

Emerging evidence from human studies places pancreatic beta cell dysfunction at the centre of early type 2 diabetes pathogenesis. While insulin resistance is a hallmark of the disease, it is insufficient insulin secretion from the beta cell that ultimately drives the transition to hyperglycaemia. Increasingly, genetic, physiological and molecular data are converging to highlight the key role of the beta cell in determining individual susceptibility to disease.

A longstanding question in diabetes research is whether beta cell dysfunction represents an initiating defect in type 2 diabetes or whether it emerges in response to insulin hypersecretion. This issue has been extensively addressed in recent years, without a clear resolution, and we refer the reader to recent reviews on the topic [1, 2].

**Fig. 1:**
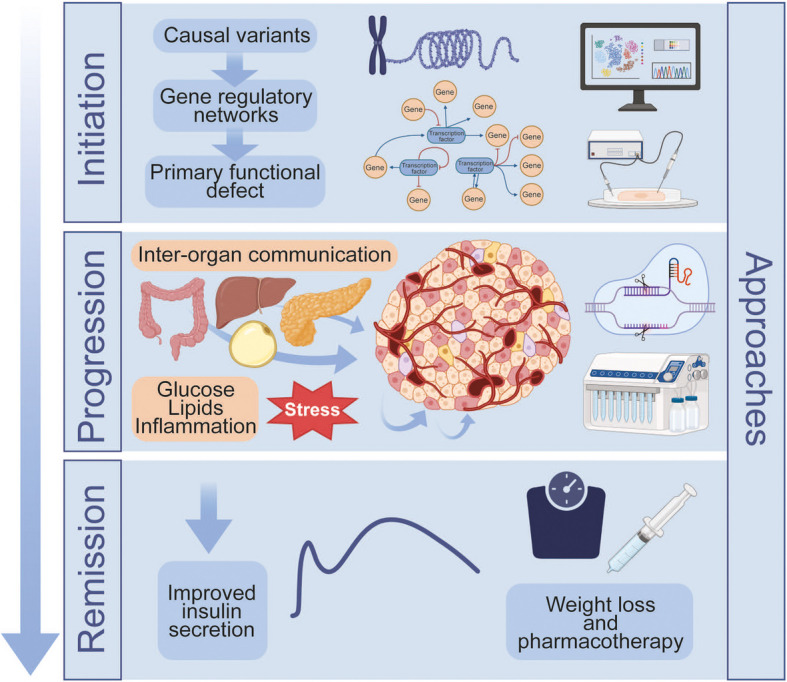
Recent developments in the role of the beta cell in the early pathogenesis, progression and remission of type 2 diabetes. The application of novel multi-omics methodologies has identified links between type 2 diabetes risk variants and gene regulatory networks in the initiation of the disease and have highlighted the role of intra-islet and inter-organ crosstalk, miRNA and beta cell heterogeneity in disease progression. Clinical data indicate that disease remission is achievable through weight loss or metabolic interventions that restore beta cell function. This figure is available as a downloadable slide

In this section we explore how human genetic studies have shaped our understanding of beta cell dysfunction in type 2 diabetes. Resources such as TIGER, which provides extensive islet expression quantitative trait loci (eQTL) data, have been instrumental in identifying novel type 2 diabetes risk genes [3]. We highlight discoveries from genome-wide association studies (GWAS) and rare variant analyses and examine how integrative functional approaches are illuminating the mechanisms by which genetic variation impairs beta cell function and contributes to disease progression (Fig. 1).

### Human genetic evidence

To date, over 500 independent loci have been associated with type 2 diabetes [4], many of which implicate genes with key roles in beta cell biology [5]. While most common variants have a modest effect size, rare coding variants identified through large-scale sequencing efforts often exhibit stronger effects and, in some cases, offer direct insight into disease mechanisms.

Recent studies have implicated several such genes in type 2 diabetes risk. *GIGYF1* (encoding GRB10 interacting GYF protein 1, a regulator of IGF signalling) is associated with an increased risk of diabetes [6]. Loss-of-function variants in *GIGYF1* are linked to reduced circulating IGF-1 levels and impaired insulin signalling, suggesting a role in maintaining systemic insulin sensitivity and metabolic homeostasis. While the precise role of GIGYF1 in islet biology remains to be fully elucidated, functional studies point toward a potential role in beta cell stress responses and survival, especially under metabolic stress. Exome sequencing has identified the mitogen-activated protein kinase gene *MAP3K15* as being involved in cellular stress response pathways. Variants in MAP3K15 have been linked to increased beta cell vulnerability in the context of metabolic and inflammatory stress [7]. *ITFG3* (encoding integrin α FG-GAP repeat containing 3 [ITFG3]), also known as *FAM234A*, has emerged as another gene of interest. Rare variants in this gene have been associated with glycaemic traits in population-based studies [8], indicating a potential role in glucose regulation. Furthermore, variants affecting ITFG3 expression in islets are associated with altered beta cell function [9].

Among the best-characterised examples of rare variant discovery translating into mechanistic insight is *SLC30A8*, which encodes the zinc transporter ZnT8. Protective loss-of-function mutations in *SLC30A8* were first identified through gene burden and population-level analyses [10]. A stop-gain variant (p.Arg138*) in the Greenlandic population was associated with markedly reduced type 2 diabetes risk, a finding replicated in the UK Biobank, in which carriers of protein-truncating variants exhibited up to a 65% lower risk of developing diabetes. Follow-up functional studies in human beta cells revealed that these variants enhance insulin secretion and improve proinsulin processing, likely by alleviating zinc-mediated inhibition of granule maturation [11]. These findings suggest that reduced ZnT8 activity may enhance beta cell efficiency under metabolic stress.

However, efforts to model these findings in vivo have highlighted substantial limitations. *Slc30a8* knockout mice display conflicting phenotypes across studies, ranging from hyperglycaemia and impaired insulin secretion [12] to protection against cytokine-induced stress and hypoxia [13]. These discrepancies underscore the challenges of modelling human genetic variation in experimental systems and emphasise the importance of context-specific follow-up [10].

Despite such complexities, integrative functional approaches are beginning to bridge the gap between genetic association and mechanism. This is especially important because most type 2 diabetes-associated variants lie in non-coding regions, complicating identification of causal variants, effector genes and relevant tissues. Tools such as CRISPR screens, chromatin conformation mapping, single-cell transcriptomics and epigenetic profiling are now enabling the dissection of regulatory networks driving beta cell dysfunction. Type 2 diabetes candidate genes, including *PAX5*, were recently validated for functional relevance in human islets, further underscoring the value of experimental follow-up in disease-relevant cell types [14].

For instance, a recent CRISPR screen in human beta cells [15] identified *CALCOCO2* (encoding calcium binding and coiled-coil domain-containing protein 2 [CALCOCO2], a selective autophagy receptor) as a regulator of insulin content. CALCOCO2 is central to mitophagy, which is essential in beta cells because of the tight coupling between mitochondrial metabolism and insulin secretion [16]. Loss of CALCOCO2 impairs mitophagic flux, promotes oxidative stress and disrupts insulin granule maturation. CALCOCO2 was previously implicated in type 2 diabetes through GWAS [17] and is now emerging as a key modulator of beta cell resilience under metabolic stress.

Complementary studies using human pluripotent stem cells have provided insights into how non-coding variants affect beta cell development. For instance, deletion of a type 2 diabetes-associated enhancer within the *ONECUT1* locus reduced the number of one cut homeobox 1 (ONECUT1)- and pancreatic and duodenal homeobox 1 (PDX1)-positive pancreatic progenitors [18], implicating this element in early beta cell differentiation. Variants in *ONECUT1* are linked to both monogenic and polygenic forms of diabetes [19].

Another study linked type 2 diabetes-associated variants in *MAP3K5* to transcriptomic changes in human islets under stress, suggesting that genetic risk factors can predispose beta cells to dysfunction in response to metabolic and inflammatory challenges [20].

Epigenetic mechanisms are also increasingly recognised as key modulators of beta cell function and contributors to type 2 diabetes risk. These include DNA methylation, histone modifications and non-coding RNAs such as miRNAs. In a recent comprehensive analysis of human islets from donors with and without type 2 diabetes, Rönn et al [21] demonstrated widespread DNA methylation changes at regulatory regions associated with beta cell function. Importantly, several of these methylation sites also showed expression quantitative trait methylation effects, providing a direct link between epigenetic variation and transcriptomic output in islets.

These advances collectively are transforming static genetic associations into dynamic insights into type 2 diabetes pathogenesis. While this section highlights genes where mechanistic insight is emerging, key loci such as *TCF7L2* remain poorly understood at the cellular level, underscoring the continued need to bridge human genetics with beta cell biology to fully elucidate pathogenesis. Validation studies are not only critical to discarding potential artefactual findings but also have begun to unravel the functional consequences of type 2 diabetes risk variants.

### Functional evidence from human studies: beyond beta cell mass

The significance of functional beta cell mass is perhaps best illustrated in prediabetes, where beta cell mass and function are often discordant. Functionally, impaired glucose tolerance (IGT), a key prediabetic state, is associated with reduced first- and second-phase insulin secretion and lower total insulin content normalised to beta cell mass [22]. Importantly, when assessed in the context of clinical outcomes, beta cell mass does not correlate with HbA_1c_ or fasting glucose. Instead, glucose-stimulated insulin secretion is markedly reduced in individuals with IGT [22], suggesting that beta cell function, rather than mass, may be the primary driver of early metabolic decline.

Analysis of donor islets in the Human Pancreas Analysis Program (HPAP) has revealed altered expression of genes involved in insulin granule docking and exocytosis in donors with IGT and type 2 diabetes, including reduced expression of *STX1A*, *VAMP2* and *UNC13A*, which are critical components of the vesicle fusion machinery [23]. These findings align with reduced first-phase insulin secretion observed clinically, supporting the idea that early defects in stimulus–secretion coupling precede measurable changes in mass.

Further, the EXODIAB biobank identified upregulation of haemostasis and complement cascade-associated proteins in islets from prediabetic donors compared with control donors, while these proteins were downregulated in overt diabetes [24]. This biphasic pattern suggests a functional compensatory phase followed by decompensation and failure. Similarly, serum proteomic analyses from the AGES-Reykjavik/AGESII cohort identified several candidate protein predictors of type 2 diabetes (IGF-binding protein 2 [IGFBP2], apolipoprotein M [APOM], inhibin βC chain [INHBC] and growth hormone receptor [GHR]), many of which are implicated in insulin signalling and metabolic stress pathways [25].

More recent data from the IMI RHAPSODY project, which integrates clinical cohorts from Europe with longitudinal proteomic and metabolomic profiling, have identified novel beta cell stress markers that precede diabetes onset [26]. For instance, elevated circulating levels of fatty acid-binding protein 4 (FABP4) and glutathione peroxidase 3 (GPX3) were associated with declining insulin secretion, independent of insulin resistance [27]. These markers may reflect oxidative stress and lipid remodelling within beta cells, both hallmarks of functional decline.

Genetic risk scores further support the central role of beta cell dysfunction in type 2 diabetes progression. Billings et al [28] analysed the Diabetes Prevention Program (DPP) for type 2 diabetes polygenic scores (pPS) mapped to five genetic clusters: beta cell dysfunction; circulating proinsulin; obesity; lipodystrophy; and liver lipid metabolism. The beta cell-specific genetic risk score, composed of 30 beta cell function SNPs, was strongly associated with increasing beta cell dysfunction from prediabetes to diabetes, reinforcing the idea that beta cell failure is a primary driver of disease progression.

Most recently, single-cell RNA-seq of human islets from non-diabetic donors, prediabetic donors and donors with type 2 diabetes has revealed progressive beta cell dedifferentiation and loss of identity markers (e.g. MafA, PDX1), coupled with increased stress response genes (e.g. *DDIT3*/*CHOP*, *TXNIP*) in type 2 diabetes islets [29]. These data support a continuum of beta cell dysfunction that is molecularly distinct from mere cell loss and highlights the importance of preserving differentiated beta cell state.

### Defining type 2 diabetes subtypes and their role in disease progression

The heterogeneity of type 2 diabetes presents an opportunity to better understand beta cell pathology across subgroups of individuals. Ahlqvist et al use six clinical variables (GADA, BMI, diabetes onset age, beta cell function, HbA_1c_ and insulin resistance) to define five subtypes of adult-onset diabetes, mapped in the ANDIS cohort [30]. These subtypes associate with unique clinical presentations and disease outcomes. The severe insulin-deficient diabetes (SIDD) subtype is characterised by high HbA_1c_, low HOMA-B and low HOMA-IR**,** resembling adult-onset type 1 diabetes but in the absence of autoantibodies. Importantly, SIDD exhibits the fastest progression to insulin dependence, suggesting a more aggressive form of beta cell failure.

## The role of the beta cell in type 2 diabetes progression

### Impact of the metabolic environment on the beta cell

#### Nutrient-induced beta cell dysfunction

Glucolipotoxicity (the deleterious effect of elevated glucose and fatty acids on beta cell function) has long been proposed to underlie the gradual deterioration of insulin secretion during the course of type 2 diabetes [31, 32] (Fig. 1). However, the extent to which glucolipotoxicity contributes to beta cell dysfunction in type 2 diabetes has been contested, largely due to the challenges in accurately determining the specific fatty acid species and concentrations that beta cells are exposed to in vivo [33]. Of particular interest in this regard, Marselli et al [34] analysed the transcriptome of non-diabetic donor islets exposed to glucolipotoxic stress and identified upregulated pathways associated with beta cell failure including the unfolded protein response (UPR) and endoplasmic reticulum (ER) stress. Then, by comparing gene sets regulated by glucolipotoxicity with those altered in type 2 diabetes, overlapping pathways were identified, including upregulation of peroxisome proliferator-activated receptor (PPAR) signalling and downregulation of beta cell function and identity, suggesting that glucolipotoxic stress contributes to beta cell dysfunction in type 2 diabetes.

Although Marselli et al [34] observed an enrichment of inflammatory and immune response pathways in islets from individuals with type 2 diabetes, only a few inflammation-related genes (notably *CXCL2*, *CXCL8* and *TRIB3*) were upregulated in islets under glucolipotoxic stress. This difference was attributed to immune cell recruitment occurring in vivo. Supporting this, Wu et al [35] described islet infiltration by CD8^+^ cytotoxic T cells and macrophages in type 2 diabetes, likely driven by chronic hyperglycaemia and hyperlipidaemia [36]. Further, Maestas et al [37] demonstrated that proinflammatory cytokines and ER stressors upregulate genes (e.g. *CIB1*) in both alpha and beta cells, with *CIB1* protecting against cytokine-induced apoptosis while regulating beta cell identity.

#### Inter-organ crosstalk

While the direct effects of hyperglycaemia and hyperlipidaemia on the beta cell have been well studied, adipose tissue, skeletal muscle, liver, intestine and the exocrine pancreas acting via tissue-specific mediators, including metabolites, proteins and miRNAs (short, non-coding RNAs that silence gene expression) indirectly impact beta cell function [38] (Fig. 1). Along these lines, Zhang et al [39] described a novel mechanism in the mouse whereby microRNA miR-27a-5p containing extracellular vesicles released from visceral adipocytes was taken up by beta cells resulting in beta cell dysfunction due to the downregulation of adenylate cyclase 1 (encoded by *Adcy1*) and L-type Ca^2+^ channel subunit α 1c (encoded by *Cacna1c* [*Cav1.2*]). In humans, serum and islet levels of miR-27a-5p increased in both obesity and type 2 diabetes and were inversely correlated with beta cell function. Additional studies describing changes in miRNA expression in human islets are described in the following section. Scheithauer et al [40] showed that increased gut Gram-negative Enterobacteriaceae species are linked to hyperglycaemia in humans and induced inflammation and dysfunction in mouse beta cells. Mechanistically, bacterial flagellin induces a proinflammatory response by activating Toll-like receptor-5 (TLR5) in macrophages, and the presence of flagellin antibodies in pancreatic biopsies of individuals with type 2 diabetes lends support to this mechanism in humans. Additionally, Kong et al [41] showed that dietary factors metabolised by gut microbes can produce trimethylamine, which is transported to the liver and converted by hepatic flavin mono-oxygenase 3 (FMO3) into trimethylamine *N*-oxide (TMAO). TMAO levels are elevated in type 2 diabetes and TMAO negatively impacts beta cell function and survival. Finally, Basile et al [42] demonstrated that, in type 2 diabetes, *CELA3B*, which encodes pancreatic elastase (PE), is upregulated in pancreatic acinar cells and the active form of PE is present in the islet microenvironment. Pathophysiological levels of PE increased apoptosis and reduced beta cell proliferation and insulin secretory capacity in human beta cell lines ex vivo. However, it remains to be determined whether PE in the type 2 diabetes islet microenvironment similarly impacts the beta cell in vivo. Mechanistically, PE interferes with extracellular matrix (ECM)–integrin mechano-signalling and the protease-activated receptor (PAR) signalling cascade. Together, these studies highlight the contribution of inter-organ crosstalk in beta cell dysfunction in type 2 diabetes.

### Mechanistic changes in the beta cell

Insight into the molecular pathogenesis of beta cell dysfunction in type 2 diabetes has benefited from a surge in studies using cutting-edge technologies to generate physiological, histological, transcriptomic, epigenomic and genomic datasets of donor islets. Many of these studies rely on the National Institute of Diabetes and Digestive and Kidney Diseases (NIDDK) Human Pancreas Analysis Program for Type 2 Diabetes (HPAP-type 2 diabetes) repository or the consolidated PanKbase (https://pankbase.org/) [43].

#### Beta cell heterogeneity

In a seminal study, Bader et al [44] demonstrated that the Wnt/planar cell polarity (PCP) pathway underlies beta cell heterogeneity and controls beta cell maturation. Since then, growing evidence suggests that functional heterogeneity among beta cells is essential for proper insulin secretion [45]. However, distinguishing transient cell states from stable subtypes remains challenging. By analysing epigenetic silencing modifications (H3K27me3), Dror et al [46] identified two beta cell subtypes (beta-high and beta-low), matching clusters previously identified in mice and humans, including beta1/beta2 from Dorrell et al [47] and INS^high/low^ from Chiou et al [48]. These subtypes were stable in culture and showed distinct cell surface marker (CD24) expression, with beta-high (CD24^+^) exhibiting increased mitochondrial mass and insulin secretion compared with beta-low (CD24^−^). Thus, it appears that stable beta cell subtypes are a hallmark of functional islets under physiological conditions.

However, caution is warranted, as isolation artefacts may influence observed heterogeneity. Kang et al [49] compared transcriptomes of cultured vs engrafted human islets and found shifts in cluster proportions, with the more mature (beta1) increased and immature (beta3) decreased in islets in vivo. This study suggests that in vitro manipulation of islets may introduce maturational defects that warrant consideration when interpreting the physiological relevance of beta cell clusters.

A recent three-dimensional, whole-organ map of the entire human islet mass, distribution and composition highlighted previously unrecognised heterogeneity in islet size and relative endocrine cell proportions, including over 50% of islets containing insulin-positive cells but no glucagon-positive cells [50]. Importantly, this study raises the possibility of differences in beta cell subtype composition of individual islets; this has been overlooked in dispersed-islet studies to date. Hence, heterogeneity in islet size and cellular composition should be taken into consideration.

How dysfunction of specialised beta cell subtypes might contribute to type 2 diabetes was addressed in a series of studies. Rubio-Navarro et al [51] identified a CD63^high^ beta cell cluster enriched for mitochondrial metabolism genes that showed enhanced function compared with CD63^low^ beta cells in transplantation studies. Interestingly, the proportion of CD63^high^ beta cells was reduced in type 2 diabetes. Using Patch-seq to simultaneously measure electrophysiological properties and the transcriptome of individual beta cells, Camunas-Soler et al [29] identified gene sets associated with functional heterogeneity in non-diabetic donors, where expression of *FAM159B* and *RBP4* correlated positively and negatively with exocytosis, respectively. Surprisingly, in type 2 diabetes, genes positively correlated with exocytosis were upregulated whereas those negatively correlated were downregulated, possibly reflecting compensatory adaptation to increased insulin demand. However, reduced exocytosis in type 2 diabetes was associated with the upregulation of immune pathway genes downstream of the transcription factor *ETV1*. Notably, downregulating *ETV1* rescued exocytosis in type 2 diabetes but not non-diabetic donor beta cells. In a multi-omics study, Wang et al [52] identified two functionally distinct beta cell subtypes: beta1, governed by *HNF1A*, *HNF4A* and *HNF4G*; and beta2, governed by *TCF4*, *NEUROD1* and *NFIA*. Although beta2 cells showed higher exocytotic capacity and increased abundance in type 2 diabetes, the secretory capacity was impaired in both subtypes. Pathway analysis identified a stress-dependent transcription factor programme (e.g. *XBP1* and *ATF6*) that was upregulated in both beta cell subtypes in type 2 diabetes. Similarly, Weng et al [53] observed *HNF1A*-driven beta cell heterogeneity in non-diabetic donors and observed reduced expression of *HNF1A* in type 2 diabetes. Using Patch-seq, a negative correlation between the hepatocyte nuclear factor 1-α (HNF1A) target gene *FXYD2* (an inhibitory subunit of the Na^+^/K^+^-ATPase) and Na^+^ influx was observed, suggesting a model whereby HNF1A facilitates membrane depolarisation and insulin secretion by upregulating sodium/potassium-transporting ATPase subunit γ (FXYD2). While further validation is needed, loss of HNF1A and FXYD2 would be expected to hyperpolarise the membrane and impair insulin secretion.

Of note, mutations in subtype-defining transcription factors, including *HNF1A*, *TCF4*, *NEUROD1* and *HNF4A*, cause MODY [52, 53], and their *cis*-regulatory sites map to type 2 diabetes risk loci. Thus, perturbed transcriptional programmes controlling beta cell identity may be causal in type 2 diabetes. Interestingly, chronic glucose/palmitate exposure deregulates *HNF1B* and *HNF4A* in human islets [34], linking glucolipotoxic stress to beta cell subtype dysfunction in type 2 diabetes.

#### miRNA

Comparison of mRNA expression levels between normal islets and islets in type 2 diabetes have provided substantial insight into mechanisms underlying the progression of the disease; however, equivalent studies regarding the expression of miRNA have been limited, despite their potential importance in type 2 diabetes pathophysiology. In a large-scale genetic study Taylor et al [54] investigated the genetic regulation of miRNAs in human islets and identified multiple miRNAs associated with HbA_1c_ levels and type 2 diabetes. Mechanistically, Ofori et al [55] showed that miR-200c is upregulated in islets of individuals with type 2 diabetes and targets several mRNAs including the transcription factor ETS translocation variant 5 (ETV5). Downregulation of *ETV5* reduces insulin secretion possibly through reduced expression of exocytotic genes. Similarly, Cheung et al [56] demonstrated that miR-125b-5p expression is upregulated by glucose in an AMP-activated protein kinase (AMPK)-dependent manner and targets the transporter of lysosomal hydrolases, cation-dependent mannose-6-phosphate receptor (M6PR), and the mitochondrial fission regulator, mitochondrial fission process protein 1 (MTFP1), to regulate organelle dynamics and impair glucose-stimulated insulin secretion. Together these studies underscore the importance of miRNAs in human beta cell function and point to their implication in type 2 diabetes.

#### Beta cell senescence and dedifferentiation

Beta cell dysfunction in diabetes may also be attributable to the accumulation of senescent, dedifferentiated and transdifferentiated cells. Chronic metabolic stress induces beta cells to adopt a senescence-associated secretory phenotype (SASP), characterised by cytokines and ECM remodelling factors, that spreads into the surrounding environment promoting beta cell failure [57]. Although an early study by Butler et al [58] demonstrated that non-hormone-expressing endocrine cells increase only modestly in islets in type 2 diabetes, by characterising the islet maturation programme at the transcriptional level during human ontogeny, Avrahami et al [59] demonstrated that a large fraction of beta cells in type 2 diabetes undergoes dedifferentiation, characterised by de-repression of immature genes. Notably, downregulation of the polycomb repressive complex (PRC2) histone methyltransferase gene *EZH1* contributes to reduced gene silencing. Wang et al [60] demonstrated that altered RNA-binding protein levels in type 2 diabetes associate with splicing deregulation, loss of beta cell maturity and transdifferentiation to an alpha cell-like fate. Deregulation of the RNA-binding proteins poly(rC)-binding protein 2 (PCBP2) and RNA-binding protein fox-1 homolog 2 (RBFOX2) similarly correlates with defective insulin secretion [61, 62].

### Sex differences

Biological sex affects type 2 diabetes traits, including adiposity, insulin resistance and insulin secretion. Indeed, ketosis-prone diabetes occurs predominantly in Black men [63], and adult-onset beta cell dysfunction due to a mutation in the MafA transcription factor is more frequent in men than in women [64]. However, most studies of beta cell dysfunction in type 2 diabetes neglect biological sex as a variable. Qadir et al [65] described sex-specific mechanistic differences in type 2 diabetes whereby mitochondrial failure drove beta cell dysfunction in women, whereas secretory pathway downregulation predominated in men. Brownrigg et al [66] identified sex-specific gene expression changes, with beta cells in female participants exhibiting greater resistance to dysfunction. Because peripheral insulin sensitivity differences could confound these observations, the authors validated in mice that female islets upregulate the UPR pathway and resist ER stress, mirroring human findings. Together, these studies underscore the importance of sex-specific analyses in type 2 diabetes research.

## The role of the beta cell in diabetes remission

If type 2 diabetes progression is associated with declining beta cell function, the restoration thereof may hold the key to diabetes remission. The term ‘type 2 diabetes remission’ refers to a sustained metabolic improvement measurable by a return of HbA_1c_ to <48 mmol/mol (<6.5%) and near normal levels of blood glucose without pharmacotherapy. Similarly, the idea of ‘beta cell remission’ refers to a state where beta cells regain sufficient insulin secretory capacity, often through weight loss or metabolic interventions that reduce cellular stress (Fig. 1).

Clinical evidence demonstrates that strategies to reduce beta cell workload preserve function and promotes remission. These data build on robust preclinical studies identifying molecular pathways for preserving beta cell function. Meanwhile, newer incretin-based therapies have been a game-changer in inducing both beta cell and type 2 diabetes remission, the basic mechanisms of which are still being actively investigated. Here we review key evidence from ex vivo and in vivo/clinical studies and discuss their implications for diabetes management.

### Glucose-lowering and beta cell rest

Beta cell rest, achieved through either glucose-lowering therapy (e.g. insulin), which reduces secretory demand via improved glucose levels, or direct beta cell suppression (e.g. diazoxide) can reduce cellular stress and preserve functional capacity. Clinical evidence demonstrates that early insulin therapy (2–5 weeks) in individuals newly diagnosed with type 2 diabetes can yield years of glycaemic control [67–70], supporting the reversibility of early beta cell dysfunction. Similarly, diazoxide improves beta cell function by preventing insulin release through K_ATP_ channel activation [71, 72], though its clinical use remains limited to hyperinsulinaemic conditions. Newer agents such as sodium–glucose cotransporter 2 inhibitors lower glucose independently of weight loss, restoring beta cell glucose sensitivity [73, 74] while reducing oxidative/ER stress. Transcriptome data [34] reveal a critical window for intervention, showing that beta cells can recover after mild metabolic stress before irreversible glucolipotoxicity occurs. Emerging human islet studies further elucidate these mechanisms, identifying key regulators of stimulus–secretion coupling including TWIK-related alkaline pH-activated K^+^ channel 1 (TALK-1) [75] and transient receptor potential cation channel subfamily M member 4 (TRPM4) [76], calcium signalling defects [77] and granule maturation via phosphatidylinositol transfer protein alpha (PITPNA) restoration [78]. These findings bridge clinical observations to molecular pathophysiology, revealing how beta cell rest interventions may restore secretory capacity.

### Reversing beta cell damage through weight loss

Like beta cell rest strategies, sustained weight loss can induce long-term diabetes remission. The DPP demonstrated that a 7% weight reduction lowered type 2 diabetes incidence by 58% overall and by 71% in participants aged >60 years, outperforming metformin [79]. Similarly, the DIADEM trial showed 60% remission rates in younger individuals (aged 18–50 years) with early intervention [80], proving that reversibility is achievable across ages.

Mechanistically, weight loss reduces liver and pancreatic fat and suppresses beta cell dedifferentiation [81, 82]. The UK DiRECT trial achieved 46% remission through dietary weight management, with some participants sustaining remission for at least 5 years [83, 84]. Responders exhibited restored glucose- and arginine-stimulated acute insulin secretion [85, 86], directly linking beta cell recovery to diabetes reversal.

Metabolic surgery offers rapid metabolic improvements. The ARMMS-T2D study pooled data from four US trials, showing superior glycaemic control and higher remission rates with bariatric surgery vs medical/lifestyle intervention over 7–12 years [87]. Benefits include reduced liver steatosis, improved adipose function and improved insulin sensitivity [88]. Notably, gastrointestinal alterations post-surgery also affect incretin secretion, bile acid metabolism, microbiome and neuronal signalling, all of which may impact beta cell function independent of weight loss. To this point, animal models have demonstrated rapid time courses of enhanced beta cell Ca^2+^ signalling and intra-islet connectivity after bariatric surgery, before weight loss occurs, which may explain acute improvements in insulin secretion [89].

### Incretins do both, very well

Incretin-based therapies have emerged as powerful tools for improving beta cell function while promoting weight loss. The glucagon-like peptide-1 (GLP-1) receptor agonist semaglutide and the dual agonist for GLP-1 and glucose-dependent insulinotropic polypeptide (GIP) receptors tirzepatide demonstrate remarkable efficacy for diabetes remission and metabolic restoration [90, 91]. Recent clinical evidence shows that retatrutide achieves up to 24% weight reduction while improving key beta cell function markers including proinsulin/C-peptide ratios, HOMA-B scores and adiponectin levels [92]. Ongoing studies are investigating whether incretins improve beta cell function through both weight-dependent and weight-independent mechanisms, including direct effects on beta cell proliferation, identity maintenance and secretory capacity.

As we advance through 2025, two key realities have become clear: first, type 2 diabetes remission is achievable through beta cell recovery; and second, multiple effective pathways exist to accomplish this. Sustained weight loss remains the most impactful approach, achievable through lifestyle modification, pharmacotherapy or metabolic surgery. While bariatric surgery continues to demonstrate excellent long-term outcomes, the emergence of incretin multi-agonists provides compelling non-surgical alternatives. As we head into the next 5–10 years of obesity and diabetes research, a priority will be to understand the molecular traits of beta cell functional recovery so as to harness these pathways for type 2 diabetes intervention.

## Conclusion

Building on decades of research, the last 5 years have seen a flurry of novel discoveries enabled mostly by three key developments: (1) multi-omics approaches that integrate genetic, epigenetic, transcriptomic and proteomic profiling alongside high-resolution imaging and functional studies; (2) collation of these datasets from a large number of type 2 diabetes human islet samples into publicly available repositories; and (3) large clinical trials investigating the therapeutic benefits of novel incretin mimetics. These advancements have opened new avenues of investigation and refined our understanding of beta cell biology in the aetiology, progression and remission of type 2 diabetes. They reinforce the critical and primary role of beta cells in disease initiation and progression, with many risk variants controlling genes that regulate beta cell development, function and stress resilience. These studies also highlight that functional defects in insulin secretion outweigh beta cell mass loss in driving disease, while emphasising the importance of intra-islet and inter-organ communication in disease progression. We now better appreciate how functional heterogeneity among beta cells influences their stress response, and how interventions that reduce secretory demand through medications or weight loss can sometimes restore normal glucose homeostasis.

Despite these advances, important challenges remain. Identification of genes and gene networks governing beta cell function and playing a role in type 2 diabetes pathogenesis by multi-omics approaches has yet to be translated to refinement of clinical practice or new therapies. Human islet models, while invaluable, have limitations: post-mortem samples often come from donors who experienced trauma or severe disease, received medications affecting function and underwent stressful isolation procedures. Promising alternative models, such as live pancreatic slices (less traumatic while preserving the native islet niche) [93], stem-cell derived beta cells and islet organoids (three-dimensional, laboratory-grown mini-organs that mimic the structure and function of native islets) [94] or highly differentiated human beta cell lines [95], may help overcome these limitations. Additional unresolved issues include the influence of sex and ethnic differences on disease mechanisms, which are still underexplored. Nonetheless, progress in understanding beta cell dysfunction in type 2 diabetes has been extraordinary and we hope that future research will bring even greater breakthroughs to benefit the 460 million people living with this devastating disease.

## Supplementary Information

Below is the link to the electronic supplementary material.

Figure slide (PPTX 666 KB)

## Abbreviations

CALCOCO2: Calcium binding and coiled-coil domain-containing protein 2
DPP: Diabetes Prevention Program
ECM: Extracellular matrix
ER: Endoplasmic reticulum
GLP-1: Glucagon-like peptide-1
GWAS: Genome-wide association studies
HNF1A: Hepatocyte nuclear factor 1-α
IGT: Impaired glucose tolerance
ITFG3: Integrin α FG-GAP repeat containing 3
PE: Pancreatic elastase
SIDD: Severe insulin-deficient diabetes
TMAO: Trimethylamine *N*-oxide
UPR: Unfolded protein response
