# Supplementary material for: The role of the beta cell in type 2 diabetes: new findings from the last 5 years
Source: Diabetologia. 2025 Aug 6;68(10):2092–103. doi: 10.1007/s00125-025-06499-z (PMC12423227; doi:10.1007/s00125-025-06499-z)
Supplement: Supplementary file 1 — Figure slide (PPTX 666 KB) [file 125_2025_6499_MOESM1_ESM.pptx]

## Slide 1
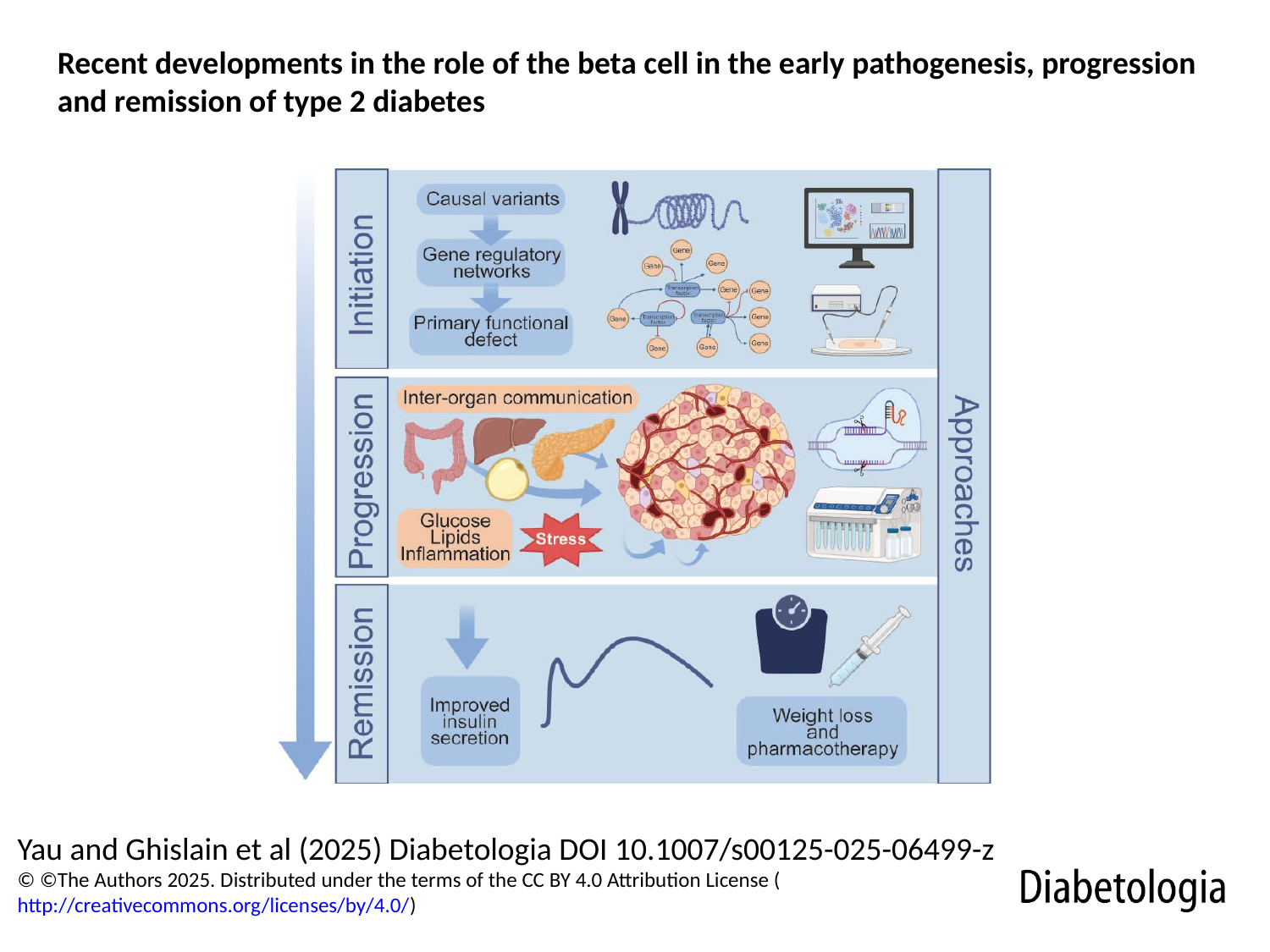

Recent developments in the role of the beta cell in the early pathogenesis, progression and remission of type 2 diabetes
Yau and Ghislain et al (2025) Diabetologia DOI 10.1007/s00125-025-06499-z
© ©The Authors 2025. Distributed under the terms of the CC BY 4.0 Attribution License (http://creativecommons.org/licenses/by/4.0/)
